# Induction of insulin secretion in engineered liver cells by nitric oxide

**DOI:** 10.1186/1472-6793-7-11

**Published:** 2007-10-17

**Authors:** Latha Muniappan, Sabire Özcan

**Affiliations:** 1 Department of Molecular and Cellular Biochemistry, College of Medicine, University of Kentucky, 741 South Limestone, BBSRB, Lexington, KY 40536, USA

## Abstract

**Background:**

Type 1 Diabetes Mellitus results from an autoimmune destruction of the pancreatic beta cells, which produce insulin. The lack of insulin leads to chronic hyperglycemia and secondary complications, such as cardiovascular disease. The currently approved clinical treatments for diabetes mellitus often fail to achieve sustained and optimal glycemic control. Therefore, there is a great interest in the development of surrogate beta cells as a treatment for type 1 diabetes. Normally, pancreatic beta cells produce and secrete insulin only in response to increased blood glucose levels. However in many cases, insulin secretion from non-beta cells engineered to produce insulin occurs in a glucose-independent manner. In the present study we engineered liver cells to produce and secrete insulin and insulin secretion can be stimulated via the nitric oxide pathway.

**Results:**

Expression of either human insulin or the beta cell specific transcription factors PDX-1, NeuroD1 and MafA in the Hepa1-6 cell line or primary liver cells via adenoviral gene transfer, results in production and secretion of insulin. Although, the secretion of insulin is not significantly increased in response to high glucose, treatment of these engineered liver cells with L-arginine stimulates insulin secretion up to three-fold. This L-arginine-mediated insulin release is dependent on the production of nitric oxide.

**Conclusion:**

Liver cells can be engineered to produce insulin and insulin secretion can be induced by treatment with L-arginine via the production of nitric oxide.

## Background

Insulin is essential in maintaining normal blood glucose levels and is produced and secreted by the beta cells of pancreas in response to increased blood glucose levels. Defects in insulin production and secretion, as observed in type 1 diabetes due to autoimmune destruction of the pancreatic beta cells, result in chronic hyperglycemia, which is responsible for most of the secondary complications associated with diabetes. Besides insulin injections, the only other option for treatment of type 1 diabetes is islet transplantation. Because of the lack of insulin production, gene therapy using surrogate beta cells is a potential approach in the treatment of Type 1 diabetes [1-3]. Delivery of insulin by gene therapy represents an attractive alternative to protein replacement therapy by potentially providing a more convenient and cost-effective delivery strategy.

Successful therapy for Type 1 diabetes requires that mature insulin be produced and secreted from surrogate beta-cells in a glucose-regulated manner. Liver appears to be an excellent surrogate organ for production of insulin, because it contains a glucose sensing machinery that is similar to pancreatic beta cells [4-7]. Liver and pancreatic beta cells both express GLUT-2 and glucokinase [7,8]. Moreover, viral gene transfer into hepatocytes is very efficient and the liver is also capable of mediating the production and release of therapeutic proteins into the systemic circulation. One major caveat of hepatic insulin production via gene therapy is that hepatocytes lack a regulated insulin secretory machinery that is present in pancreatic beta cells. Several different viral and non-viral vectors are available to target insulin expression to liver [9-11].

Several studies indicate that liver cells can be engineered to secrete biologically active insulin [12-14]. Furthermore, it has been demonstrated that introduction of the beta-cell specific transcription factors PDX-1 and NeuroD1 into hepatocytes ameliorates streptozotocin-induced hyperlgycemia in mice [15-17]. In a recent study, hepatocytes transfected with NeuroD1 and betacellulin, a β-cell growth factor, have been shown to produce insulin and to normalize blood glucose levels in streptozotocin-induced diabetic mice [18].

Changes in blood glucose levels directly control insulin secretion and also modulate the insulin-releasing effects of other secretagogues in pancreatic beta cells [19]. In addition to insulin secretion, increases in blood glucose levels also regulate insulin gene transcription by modulating the function of three beta cell specific transcription factors known as PDX-1, MafA and NeuroD1. Defects in these transcription factors have been associated with decreased insulin production and hyperglycemia [20,21].

In this study we demonstrate that the expression of human insulin or the beta-cell specific transcription factors PDX-1, MafA and NeuroD1 in the Hepa1-6 liver cell line or in primary liver cells using adenoviral gene transfer, results in production and secretion of insulin. Furthermore, we show that insulin secretion from these engineered liver cells is stimulated by treatment with L-arginine via the nitric oxide pathway. L-arginine potentiates insulin secretion also in other cell lines such as fibroblasts and cervical carcinoma cells incubated with an adenovirus containing the human insulin cDNA. This suggests that L-arginine stimulates protein secretion in various cell types via the synthesis of nitric oxide. The regulation of protein secretion by nitric oxide may be useful in the engineering of surrogate beta cells for the treatment of type 1 diabetes.

## Results

### Hepa1-6 liver cells transfected with an adenoviral vector containing human insulin are able to produce and secrete insulin

**Figure 1:**
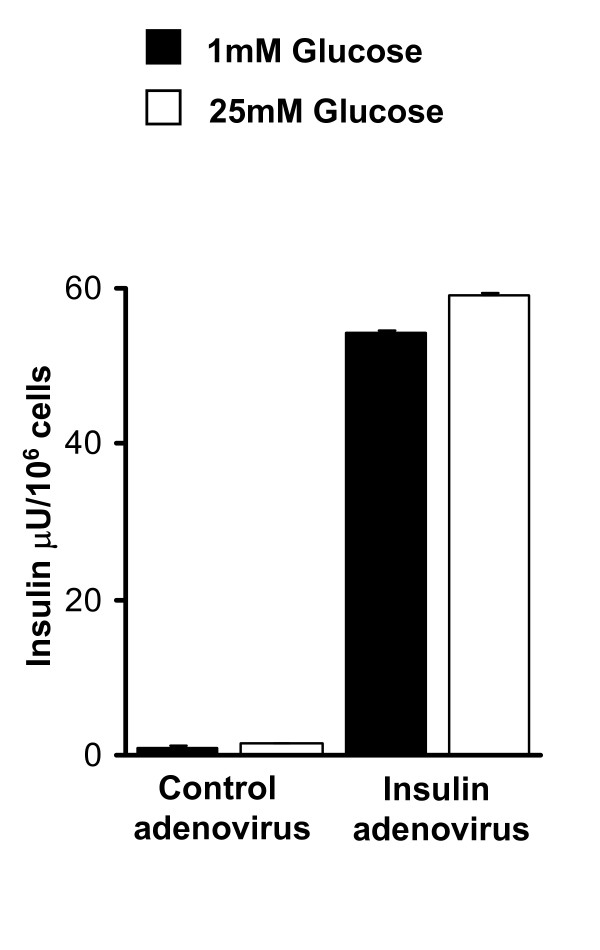
**Hepa1-6 cells expressing the human insulin gene produce and secrete insulin**. Comparison of insulin secretion from Hepa1-6 cells infected with human insulin or control adenovirus, after treatment with 1 or 25 mM glucose for 1 h. Total insulin secretion is expressed as μU/ml * 10^6 ^cells.

To test whether liver cells can be engineered to produce and secrete insulin in a regulated manner, we have incubated the Hepa1-6 liver cell line with an adenovirus expressing human insulin. Liver cells incubated with the human insulin adenovirus produced detectable amounts of insulin compared to the GFP only control virus, as demonstrated by immunostaining with an insulin antibody (Additional File 1). Next we quantified the amount of insulin secreted and the glucose-dependency of insulin secretion after treatment with 1 or 25 mM glucose using an insulin ELISA kit. The total amount of insulin secreted was about 60 μU/10^6 ^cells and the amount of insulin secreted in response to high glucose was not significantly higher than insulin secretion at 1 mM glucose (Fig. 1).

### L-Arginine induces insulin secretion in the Hepa1-6 liver cell line expressing human insulin via the nitric oxide pathway

**Figure 2:**
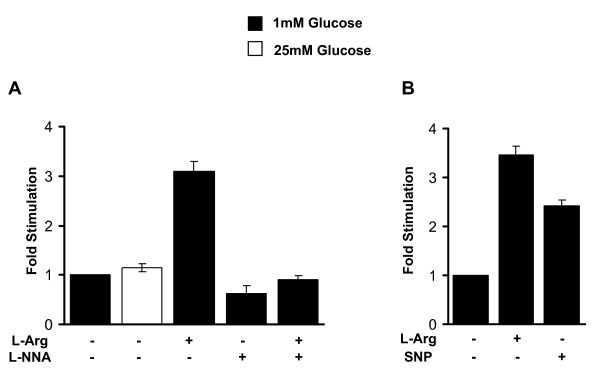
**L-arginine stimulates insulin secretion in Hepa1-6 cells expressing human insulin via the nitric oxide pathway**. A. Hepa1-6 cells incubated with the human insulin adenovirus were transferred to 1 mM glucose with or without 20 mM L-arginine in the presence or absence of the NOS inhibitor L-NNA (100 μM) for 1 h. The amount of secreted insulin in the medium was determined and is expressed as fold difference, where insulin secretion on 1 mM glucose was set as 1-fold. Values are expressed as means ± SD for n = 5 in each group. B. Effect of NO donor sodium nitroprusside (SNP) on insulin secretion in Hepa1-6 cells. Insulin secretion in Hepa1-6 cells incubated with the human insulin adenovirus was measured after incubation of cells with 1 mM glucose, with or without 20 mM L-arginine or 100 μM SNP for 1 h (n = 3).

Since L-arginine acts as a potentiator of insulin secretion in pancreatic beta cells, we tested the effect of L-arginine on insulin release in Hepa1-6 liver cells expressing human insulin. Treatment with 20 mM L-arginine for 1 h in the presence of 1 mM glucose increased insulin secretion over 3-fold (Fig. 2A). L-arginine has been reported to act via the nitric oxide pathway and therefore we determined whether the production of nitric oxide (NO) was responsible for the increase in insulin secretion in Hepa1-6 cells expressing human insulin. For this purpose, we treated the Hepa1-6 cells expressing human insulin or GFP as control with 100 μM L-NNA, an inhibitor of nitric oxide synthase (NOS) in the presence or absence of L-arginine (Fig. 2A). Treatment with the general NOS inhibitor L-NNA for 1 h abolished the L-arginine-induced insulin secretion in Hepa1-6 cells expressing human insulin (Fig. 2A). Treatment with L-NNA also inhibited basal insulin secretion, suggesting the idea that NO is responsible for some of the observed basal secretion of insulin.

Next, we tested whether the NO donor sodium nitroprusside (SNP) can induce insulin secretion similar to L-arginine. For this purpose, Hepa1-6 liver cells were treated with 100 μM SNP in the presence of 1 mM glucose for 1 h. Like L-arginine, SNP was also able to enhance insulin secretion (Fig. 2B), indicating that production of NO stimulates insulin secretion. Both, basal and L-arginine induced insulin secretion was dependent on the presence of extracellular calcium (Additional File 2A). Hepa1-6 cells incubated with KRB buffer lacking calcium displayed a 2-fold reduction in insulin secretion independent of the presence of L-arginine (Additional File 2A). Furthermore, inhibition of calcium channels using nifedipine abolished both basal and L-arginine stimulated insulin release (Additional File 2A).

Insulin secretion in pancreatic beta cells is mediated by regulated exocytosis in which the insulin containing granules fuse with the plasma membrane [22,23]. However, liver cells do not have a regulated exocytosis pathway and secretion in liver cells occurs via the constitutive secretory pathway. To test the effect of L-arginine on constitutive secretion in liver cells, we have treated Hepa1-6 cells expressing human insulin with brefeldin A (BFA), which blocks the constitutive secretion pathway by inhibiting the transport of proteins into the Golgi apparatus [24]. Treatment with brefeldin A blocked both basal and L-arginine stimulated insulin secretion in Hepa1-6 cells (Additional File 2B), but did not effect the secretion of insulin from MIN6 insulinoma cells, which occurs via regulated exocytosis from insulin granules (Additional File 2C).

### L-Arginine stimulates insulin secretion in various non-beta cell lines incubated with the human insulin adenovirus

**Figure 3:**
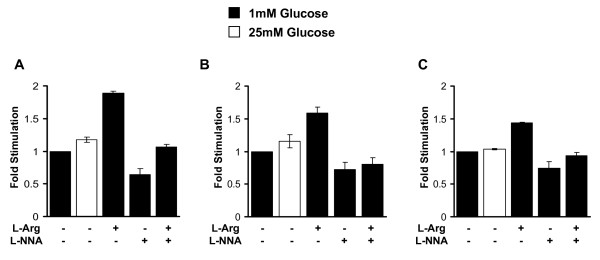
**L-arginine stimulates insulin secretion in various cell lines incubated with the human insulin adenovirus**. A. HepG2, B. NIH 3T3, and C. HeLa cells incubated with the human insulin adenovirus were transferred to 1 mM glucose overnight and washed with KRB buffer. After addition of 25 mM glucose or 1 mM glucose with or without 20 mM L-arginine and 100 μM L-NNA, insulin release was measured in the medium and is expressed as fold-difference over insulin secretion at 1 mM glucose.

To determine whether the stimulatory effect of L-arginine on insulin secretion is specific to liver cells or not, we incubated the following cell lines with the human insulin adenovirus: HepG2 (human liver), NIH3T3 (mouse fibroblast) and HeLa (human cervical carcinoma). All three cell lines were able to produce and secrete insulin. However insulin secretion was highest in the HepG2 cell line and lowest in the HeLa cell line (Additional File 3). Treatment with 20 mM L-arginine for 1 h stimulated insulin secretion in every cell line tested to various degrees (Fig 3A–C). Addition of L-arginine enhanced insulin secretion by about 2-fold in the human liver cell line HepG2 (Fig. 3A), while insulin secretion was less than 2-fold in the fibroblast cell line NIH3T3 and human cervical carcinoma (Fig. 3B &3C). In every cell line tested, stimulation of insulin secretion by L-arginine was dependent on the production of nitric oxide, since addition of L-NNA an inhibitor of nitric oxide synthase abolished L-arginine mediated insulin secretion.

### Hepa1-6 liver cells expressing beta-cell specific transcription factors produce and secrete insulin

**Figure 4:**
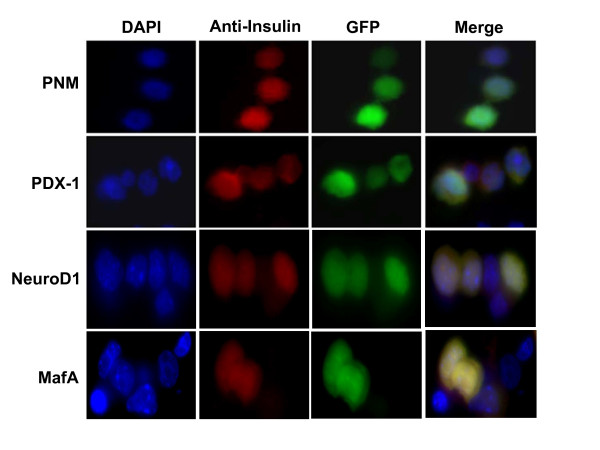
**Hepa1-6 cells expressing PDX-1, NeuroD1 and MafA produce insulin**. Hepa1-6 cells incubated with PDX-1, NeuroD1, or MafA adenoviruses separately or with all three at the same time (PNM) were immunostained for mouse insulin using a specific antibody (red). Cell nuclei were stained with DAPI (blue). Infected cells were identified by green fluorescence of GFP.

**Figure 5:**
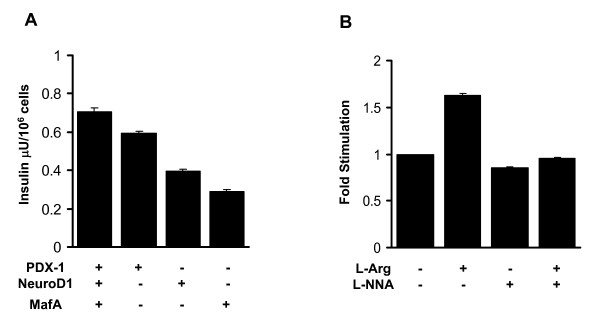
**Insulin secretion in Hepa1-6 cells expressing the beta-cell specific transcription factors is increased by treatment with L-arginine**. A. Insulin secretion in Hepa1-6 cells expressing PDX-1, NeuroD1 or MafA individually or in combination was determined using a mouse insulin ELISA kit. After incubation with the corresponding adenoviruses, the cells were incubated first over night with 1 mM glucose and then transferred to KRB buffer containing 25 mM glucose. Total insulin secretion is expressed as μU/ml * 10^6 ^cells. B. Hepa1-6 cells expressing all three transcription factors, PDX-1, NeuroD1 and MafA were incubated for 1 h in KRB buffer containing 1 mM glucose with or without 20 mM L-arginine and 100 μM L-NNA. The amount insulin in the media was quantified by an insulin ELISA assay. The data are averages of five (n = 5) independent experiments.

It has been previously shown that the beta-cell specific transcription factors such as PDX-1, NeuroD1 and MafA are able to induce insulin gene transcription in non-beta cells. Therefore, we introduced PDX-1, NeuroD1 and MafA using adenoviral gene transfer into the Hepa1-6 liver cell line and determined their effects on insulin production and secretion from liver cells. As demonstrated in Fig. 4, expression of all three beta-cell transcription factors in combination as well as individually induces insulin production in Hepa1-6 cells compared to cells expressing only GFP as control. Insulin secretion was highest in Hepa1-6 cells expressing PDX-1 and lowest in Hepa1-6 cells expressing MafA (Fig. 5A). Insulin secretion in Hepa1-6 cells expressing all three transcription factors at the same time was increased about 1.7-fold by addition of 20 mM L-arginine (Fig. 5B). L-Arginine stimulated insulin secretion was inhibited by addition of 100 μM L-NNA, which inhibits NOS (Fig. 5B).

### L-arginine induces insulin secretion in primary rat liver cells expressing human insulin

**Figure 6:**
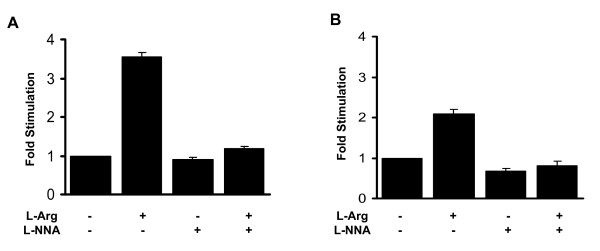
**Treatment with L-arginine induces insulin secretion in primary hepatocytes expressing human insulin or the beta-cell specific transcription factors**. Primary rat hepatocytes were incubated either with the human insulin (panel A) or a combination of PDX-1, NeuroD1 and MafA adenoviruses (panel B). After incubation on 1 mM glucose for about 16 h, the cells were transferred to KRB buffer containing 1 mM glucose in the presence or absence of 20 mM L-arginine and 100 μM L-NNA. Insulin secretion in the media was measured in three independent experiments and is expressed as fold difference of insulin secretion of 1 mM glucose incubated cells.

All of our initial experiments were carried out using the hepatoma carcinoma cell line Hepa1-6. Thus, we next confirmed the effects of L-arginine on insulin secretion in primary rat liver cells expressing human insulin. For this purpose, primary rat liver cells were cultured and incubated with an adenovirus containing human insulin (Additional File 4A). Insulin secretion from primary hepatocytes was comparable to insulin secretion from the Hepa1-6 cell line expressing human insulin and was slightly increased on high glucose compared to low glucose (Additional File 4B). As observed with the Hepa1-6 cell line, secretion of insulin was more than 3-fold higher when primary rat liver cells expressing human insulin were incubated with 1 mM glucose in the presence of 20 mM L-arginine (Fig. 6A). The L-arginine mediated increase in insulin secretion was abolished after treatment with 100 μM L-NNA (Fig. 6A), consistent with the idea that L-arginine stimulates insulin secretion via the nitric oxide pathway.

### Primary rat liver cells expressing the beta-cell specific transcription factors PDX-1, NeuroD1 and MafA produce and secrete insulin

In addition to the human insulin adenovirus, we incubated primary rat liver cells with a combination of PDX-1, NeuroD1 and MafA adenoviruses. The expression of these transcription factors in primary liver cells was confirmed by western blotting (Additional File 5A). We found that insulin secretion in primary rat liver cells expressing the transcription factors was only 4% of that observed in primary hepatocytes expressing human insulin (Additional File 5B). Insulin secretion was also slightly increased by high glucose (Additional File 5B). Incubation of these cells with 20 mM L-arginine in the presence of 1 mM glucose increased insulin secretion by 2-fold (Fig. 6B). The observed increase in insulin secretion was diminished after addition of the NOS inhibitor L-NNA (Fig. 6B).

## Discussion

In this study, we have demonstrated that expression of human insulin or beta cell specific transcription factors in the Hepa1-6 liver cell line or primary rat hepatocytes results in production and secretion of insulin. However, the secretion of insulin from these engineered cells was not very responsive to changes in glucose levels. Furthermore, we have expressed human insulin also in various cell lines such as HepG2 (human hepatoma), NIH3T3 (mouse fibroblast) and HeLa (human cervical cancer) cells. All of these cell lines were able to produce and secrete insulin.

L-arginine has been found to stimulate insulin secretion in pancreatic beta cells [25-27]. Therefore, we determined the effect of L-arginine on insulin secretion from the engineered Hepa1-6 cell line or primary hepatocytes and found that L-arginine stimulates insulin secretion up to 3-fold in these engineered tissues. The L-arginine mediated enhancement of insulin secretion was via the production of NO, since inhibition of nitric oxide synthase (NOS) abolished this effect. Furthermore, treatment with sodium nitroprusside, which is an NO donor stimulated insulin secretion similar to L-arginine.

Insulin secretion from Hepa1-6 cells expressing human insulin depends on the presence of calcium. Lack of calcium or inhibition of the calcium channels with nifedipine abolishes both basal and L-arginine stimulated insulin secretion. Brefeldin A (BFA) is an agent that blocks the transport of proinsulin to the trans-Golgi network, but has no effect on insulin secretion via granule exocytosis [24]. Addition of BFA to Hepa1-6 cells expressing human insulin blocked the secretion of insulin, while it had no effect on glucose-induced insulin secretion from the pancreatic beta cell line MIN6. This indicates that insulin secretion from Hepa1-6 cells expressing human insulin is due to constitutive secretion, while secretion of insulin in pancreatic beta cells occurs via the regulated exocytotic pathway as expected. Furthermore, L-arginine-mediated insulin secretion was also inhibited by BFA, suggesting that L-arginine stimulates insulin secretion via the nitric oxide pathway by enhancing the constitutive protein secretion pathway.

The exact mechanism(s) by which NO produced from L-arginine stimulates insulin secretion from liver cells is not known. Since liver cells were exposed to L-arginine only for 1 h, the observed enhancement of insulin secretion is unlikely due to transcriptional effects. There was no difference in insulin mRNA levels in cells treated with L-arginine for 1 h compared to untreated cells (data not shown). NO has been previously reported to stimulate synaptic vesicle exocytosis. Synaptic proteins such as SNAP 25, syntaxin and VAMP are known to be involved in NO mediated secretion [28]. It has been reported that NO is capable of S-nitrosylation of neuronal proteins including SNAP-25 and NSF [29]. NO has been also shown to associate with SNARE proteins, which play a main role in the secretory pathway [30]. We propose that the production of NO from L-arginine enhances secretion in liver by either nitrosylation of secretory proteins such as SNAP-25 or by direct interaction of NO with the secretory pathway.

## Conclusion

The presented data indicate that liver cells and other non-beta cells can be engineered to produce and secrete insulin. Although insulin secretion from these engineered cells is not very responsive to changes in glucose levels, addition of L-arginine stimulates insulin secretion up to 3-fold via the nitric oxide pathway. Stimulation of insulin secretion from surrogate beta cells via the production of NO could provide a potential therapy for the treatment of type 1 diabetes. Instead of glucose, engineered liver cells can be induced to secrete insulin using nitric oxide precursors such as nitroprusside. Since nitric oxide is very unstable, insulin secretion stimulated by nitric oxide would be transient and may avoid hypoglycemia as observed by constitutive secretion of insulin from engineered cells. Further studies will investigate the molecular basis of stimulation of insulin secretion by L-arginine in surrogate β cells, which will be a useful step in the development of insulin-replacement therapies of diabetes. Future experiments will also determine whether L-arginine can stimulate insulin secretion and thereby correct hyperglycemia in diabetic animals expressing insulin in liver.

## Methods

### Chemicals and reagents

Materials used are Waymouth's MB 752/1 medium (Invitrogen), Matrigel (BD Biosciences), insulin ultrasensitive ELISA kit (Mercodia); L-NNA (*N-*G-nitro-l-arginine, a nitric oxide synthase inhibitor) (Sigma), and Brefeldin A (Chemicon). All of the other chemicals were from Sigma Chemicals unless otherwise noted.

### Construction of the adenoviruses expressing human insulin, PDX-1, NeuroD1 and MafA

Recombinant adenoviruses expressing human insulin [31], PDX-1, NeuroD1 and MafA were prepared using the pAdEasy system [32,33] under the control of human cytomegalovirus (CMV) promoter. As a negative control, we used the empty adenoviral vector pAdTrackCMV expressing GFP. After subcloning of human insulin, PDX-1, NeuroD1 and MafA into the pAdTrackCMV vector, the vector was linearized and co-transformed into the bacterial strain BJ5183 with the adenoviral backbone plasmid pAdEasy-1. The recombinant adenoviral vectors were obtained by homologous recombination of pAdTrackCMV with pAdEasy-1 and the resultant plasmids were then re-transformed and amplified in DH5α cells. The obtained plasmids were linearized with Pac I and then transfected into the adenovirus packaging cell line HEK 293 by electroporation at 400 V and 500-μF using the GenePulser II electroporator (Biorad). The electroporation was carried out using a cell concentration of 2 × 10^6 ^cells per cuvette in DMEM (Cellgro) without serum. The cells were harvested fourteen to fifteen days after transfection, when 70–80% of the cells expressed Green Fluorescent Protein (GFP). The negative control adenovirus expressing only GFP was prepared in the same manner. After lysis of the HEK293 cells by freezing and thawing, the cell supernatant containing the viruses was collected and used for further amplification of the recombinant adenoviruses. The efficiency of adenoviral infection was determined by the ratio of cells expressing GFP.

### Isolation and culturing of adult rat hepatocytes

Animals were kept under standard conditions in the animal facility and had free access to food and water. This study was approved by the Institutional Animal Care and Use Committee (IACUC), University of Kentucky. The tissue culture dishes were treated with Matrigel (6.3 mg/ml) as described previously [34]. Hepatocytes were isolated from male Sprague-Dawley rats (180–200 g) (Harlan, Inc. Indianapolis, IN). For the isolation of primary hepatocytes, rats were anesthetized by intraperitoneal injection of sodium pentobarbitone. After in situ collagenase perfusion, the cells (1 × 10^6^/plate; viability > 80%) were plated in 3 ml of Waymouth's medium. Cultures were maintained for two days at 37°C in a 5% CO_2 _atmosphere and the medium was replaced every 24 h, starting 3 h after plating. Primary hepatocytes were incubated with adenoviruses expressing human insulin, PDX-1, NeuroD1 and MafA, and GFP as control.

### Cell culture conditions

Hepa1-6 cells (mouse hepatocyte cell line) were cultured in a humidified atmosphere at 37°C with 5% CO_2_, and grown in DMEM (Dulbecco's modified Eagle's medium) containing 10% (v/v) heat-inactivated fetal bovine serum, 25 mM glucose, 100 U/ml penicillin, and 100 mg/ml streptomycin [35].

### Insulin-ELISA assay

To quantify the amount of insulin secreted, hepatocytes were grown on a 6-well dish (about 1 × 10^6 ^cells) and incubated over night with various adenoviruses in DMEM with 10% FBS. After this incubation period, the cells were washed twice with 1×PBS and incubated for 14–16 h with 1 mM glucose without FBS. After washing the cells three times with KRB buffer (119 mM NaCl, 4.7 mM KCl, 2.5 mM CaCl2, 1.2 mM MgSO4, 1.2 mM KH2PO4, 25 mM NaHCO3, 10 mM Hepes, pH 7.4, and 0.1 g BSA), the cells were incubated in 1 ml pre-warmed KRB buffer containing 1 mM or 25 mM glucose with or without 20 mM L-arginine in the absence or presence of various compounds for 1 h at 37°C. The cell culture media (total of 1 ml) was collected and used to measure the levels of insulin released with an insulin ELISA kit (Mercodia). The amount of secreted insulin is given in μU per 1 × 10^6 ^cells. Fold stimulation refers to insulin secretion after various treatments compared to insulin secretion in 1 mM glucose treated cells, which was set as 1-fold. Values are expressed as means ± SD of data obtained from three to five independent experiments (n = 3 to 5) in duplicates.

### Western blotting

Protein extracts from hepatocytes infected with PDX-1, NeuroD1, MafA and GFP adenoviruses were blotted with PDX-1 (a gift from Dr. Chris Wright, Vanderbilt University), NeuroD1 (Santa Cruz Biotechnology), MafA (Calbiochem) and GFP (Clontech Laboratories) antibodies. Proteins were visualized by enhanced chemiluminescence (ECL) western blotting detection kit (Amersham Bioscience).

## Abbreviations

**L-Arg**, L-Arginine; **GFP**, Green Fluorescent Protein; **NO**, Nitric oxide; **NOS**, nitric oxide synthase; **L-NNA**, N^G^- nitro-L-Arginine; **NeuroD1**, Neurogenic Differentiation 1; **PDX-1**, Pancreatic Duodenum Homeobox protein-1; **MafA**, v-maf musculoaponeurotic fibrosarcoma oncogene homolog A

## Competing interests

The author(s) declare that they have no competing interests.

## Authors' contributions

LM performed all of the experiments, data quantification and drafting of the manuscript. SÖ substantially contributed to the conception and design of the experiments, revised the manuscript critically for important intellectual content. Both authors read and approved the final manuscript.

## Supplementary Material

Additional file 1 **Hepa1-6 cells incubated with an adenovirus containing human insulin produce insulin**. Hepa1-6 cells infected with the human insulin or control adenovirus, were immunostained for human insulin using a specific antibody (red). Cell nuclei were stained with DAPI (blue). Both, the human insulin and the control virus express GFP, which makes it easy to detect the infected cells by their green fluorescence. Click here for file

Additional file 2 **Insulin secretion from Hepa1-6 cells requires calcium and is inhibited by brefeldin A (BFA)**. A. Hepa1-6 cells expressing human insulin were first grown overnight with 1 mM glucose and afterwards incubated in KRB buffer with or without calcium and 20 mM L-arginine in the presence or absence of 10 μM nifedipine for 1 h. The amount of insulin in the media was determined using an insulin ELISA kit (n = 3). Hepa1-6 cells expressing human insulin (panel B) and the mouse insulinoma MIN6 cells (panel C) were incubated over night with 1 mM glucose and next day transferred to KRB buffer containing 1 mM glucose in the presence or absence of BFA (10 μg/ml) and 20 mM L-arginine. After 1 h incubation, insulin levels in the media were measured using the human and mouse insulin ELISA kit, respectively (n = 3). Click here for file

Additional file 3 **HepG2, NIH3T3, and HeLa cells incubated with the human insulin adenovirus produce and release insulin**. Insulin secretion in HepG2, NIH3T3, and HeLa cells was determined using a human insulin ELISA kit. After incubation with the human insulin adenovirus, the various cell lines were incubated first over night with 1 mM glucose and then transferred to KRB buffer containing 1 mM glucose. Total insulin secretion is expressed as μU/ml * 10^6 ^cells. Click here for file

Additional file 4 **Primary hepatocytes infected with a human insulin adenovirus produce and release insulin**. A. Cultured primary liver cells were incubated with the human insulin virus at an MOI 1:10 and after incubation for 48 h, the infection efficiency was determined by detection of GFP expression. B. Insulin secretion in cultured primary liver cells expressing the human insulin was determined after incubation with 1 mM glucose over night and transfer of cells to KRB buffer containing 1 or 25 mM glucose for 1 h (n = 3). Click here for file

Additional file 5 **Cultured primary liver cells infected with the beta-cell specific transcription factors PDX-1, NeuroD1 and MafA produce and secrete insulin**. A. Western blot analysis of PDX-1, NeuroD1 and MafA expression in primary liver cells incubated with a combination of adenoviruses containing the three transcription factors. As a negative control, primary liver cells were incubated with an adenovirus expressing only GFP. B, Insulin secretion in cultured primary liver cells expressing PDX-1, NeuroD1 and MafA was determined after incubation with 1 mM glucose over night and transfer of cells to KRB buffer containing 1 or 25 mM glucose for 1 h (n = 3). Click here for file
