# Supplementary figures and images for: Induction of insulin secretion in engineered liver cells by nitric oxide
Source: BMC Physiol. 2007 Oct 17;7:11. doi: 10.1186/1472-6793-7-11 (PMC2121102; doi:10.1186/1472-6793-7-11)

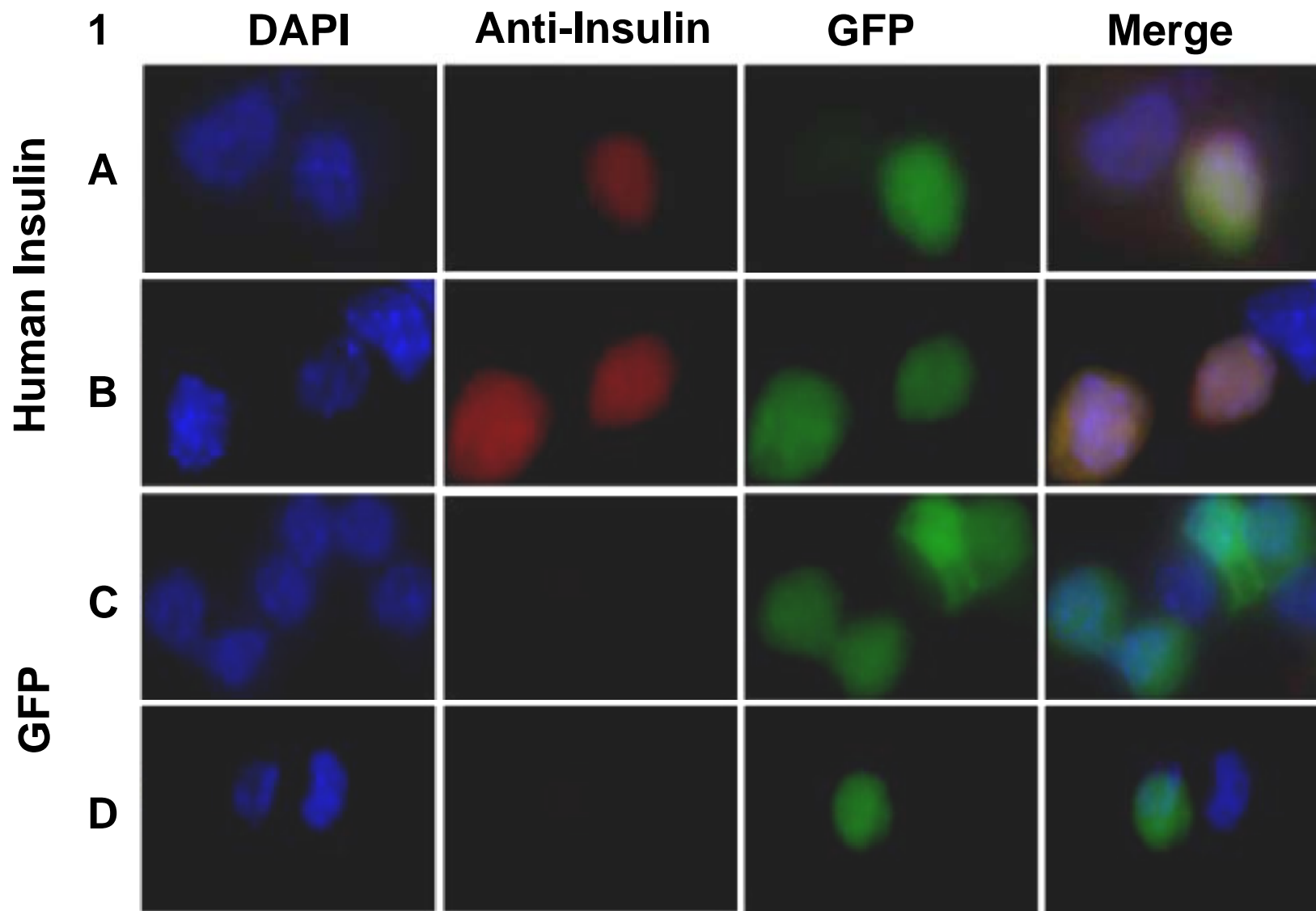

Supplement: Additional file 1 — Hepa1-6 cells incubated with an adenovirus containing human insulin produce insulin. Hepa1-6 cells infected with the human insulin or control adenovirus, were immunostained for human insulin using a specific antibody (red). Cell nuclei were stained with DAPI (blue). Both, the human insulin and the control virus express GFP, which makes it easy to detect the infected cells by their green fluorescence. [file 1472-6793-7-11-S1.pdf]

**2A**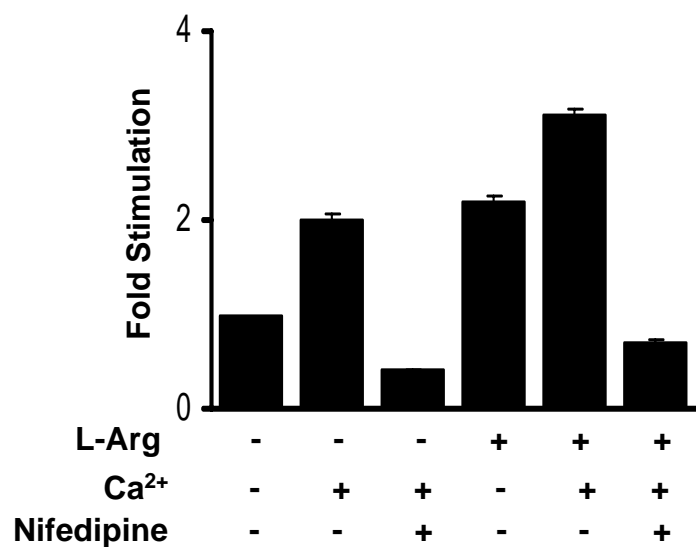**2B**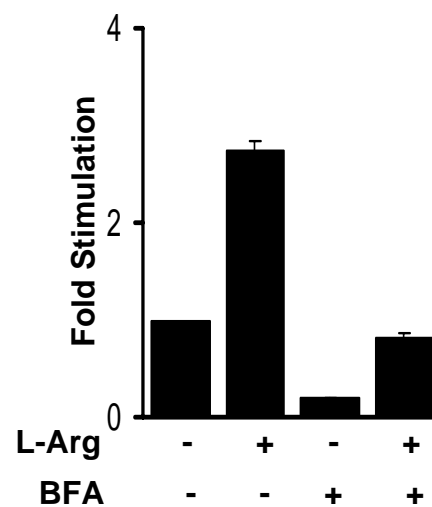**2C**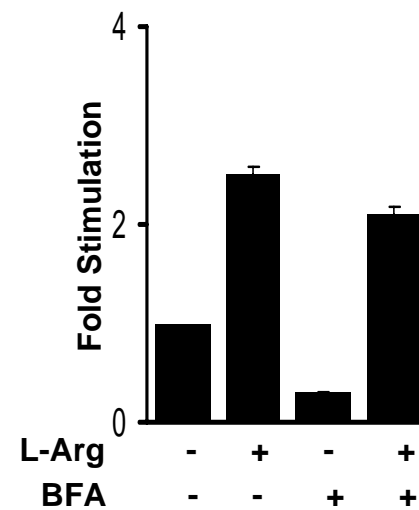

Supplement: Additional file 2 — Insulin secretion from Hepa1-6 cells requires calcium and is inhibited by brefeldin A (BFA). A. Hepa1-6 cells expressing human insulin were first grown overnight with 1 mM glucose and afterwards incubated in KRB buffer with or without calcium and 20 mM L-arginine in the presence or absence of 10 μM nifedipine for 1 h. The amount of insulin in the media was determined using an insulin ELISA kit (n = 3). Hepa1-6 cells expressing human insulin (panel B) and the mouse insulinoma MIN6 cells (panel C) were incubated over night with 1 mM glucose and next day transferred to KRB buffer containing 1 mM glucose in the presence or absence of BFA (10 μg/ml) and 20 mM L-arginine. After 1 h incubation, insulin levels in the media were measured using the human and mouse insulin ELISA kit, respectively (n = 3). [file 1472-6793-7-11-S2.pdf]

3

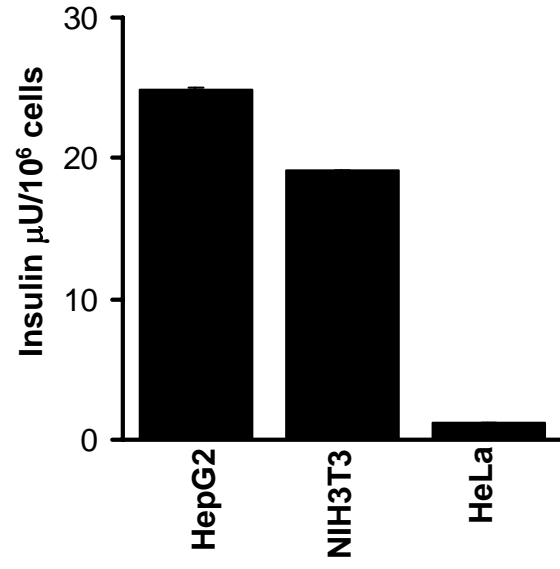

Supplement: Additional file 3 — HepG2, NIH3T3, and HeLa cells incubated with the human insulin adenovirus produce and release insulin. Insulin secretion in HepG2, NIH3T3, and HeLa cells was determined using a human insulin ELISA kit. After incubation with the human insulin adenovirus, the various cell lines were incubated first over night with 1 mM glucose and then transferred to KRB buffer containing 1 mM glucose. Total insulin secretion is expressed as μU/ml * 106 cells. [file 1472-6793-7-11-S3.pdf]

4A

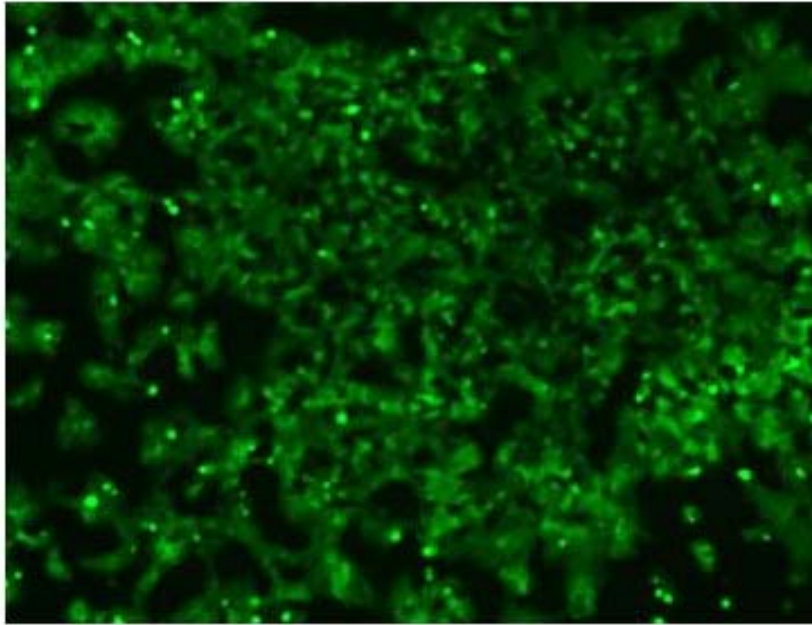

4B

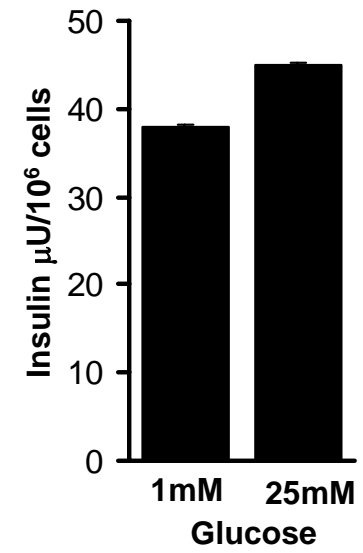

Supplement: Additional file 4 — Primary hepatocytes infected with a human insulin adenovirus produce and release insulin. A. Cultured primary liver cells were incubated with the human insulin virus at an MOI 1:10 and after incubation for 48 h, the infection efficiency was determined by detection of GFP expression. B. Insulin secretion in cultured primary liver cells expressing the human insulin was determined after incubation with 1 mM glucose over night and transfer of cells to KRB buffer containing 1 or 25 mM glucose for 1 h (n = 3). [file 1472-6793-7-11-S4.pdf]

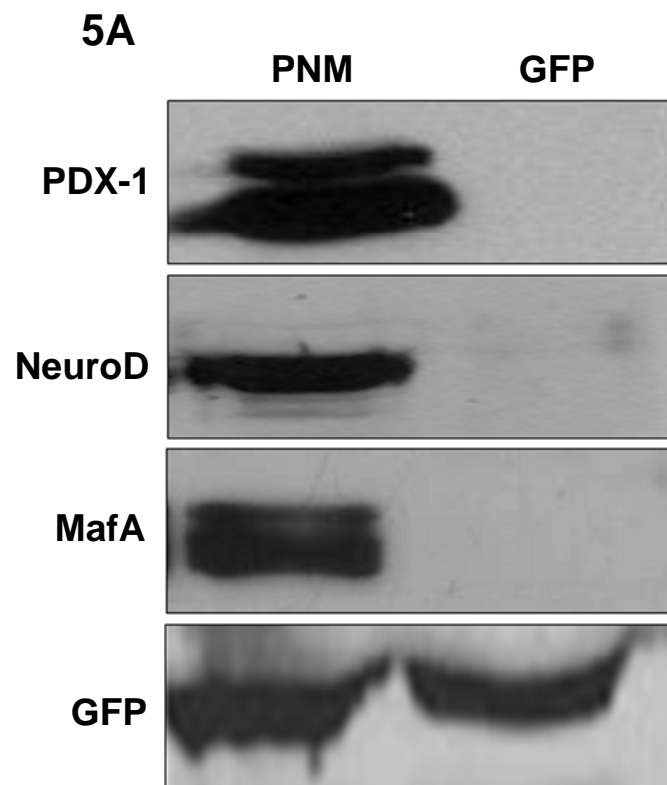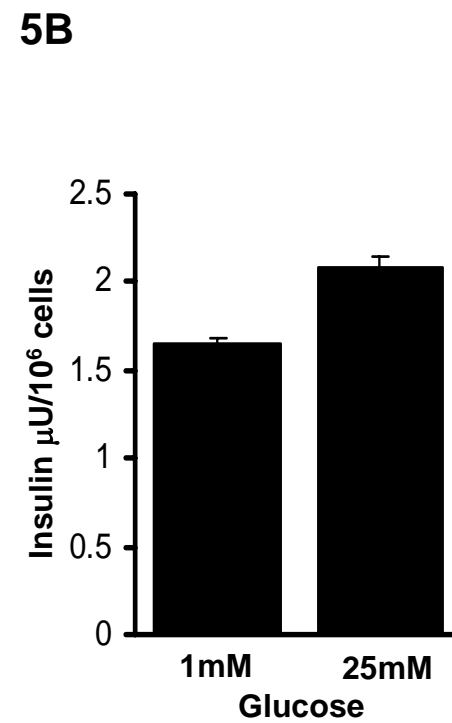

Supplement: Additional file 5 — Cultured primary liver cells infected with the beta-cell specific transcription factors PDX-1, NeuroD1 and MafA produce and secrete insulin. A. Western blot analysis of PDX-1, NeuroD1 and MafA expression in primary liver cells incubated with a combination of adenoviruses containing the three transcription factors. As a negative control, primary liver cells were incubated with an adenovirus expressing only GFP. B, Insulin secretion in cultured primary liver cells expressing PDX-1, NeuroD1 and MafA was determined after incubation with 1 mM glucose over night and transfer of cells to KRB buffer containing 1 or 25 mM glucose for 1 h (n = 3). [file 1472-6793-7-11-S5.pdf]
